# Not all days are equal after preterm prelabor rupture of membranes: a time-dependent analysis of adverse perinatal outcomes

**DOI:** 10.1186/s12884-026-10211-3

**Published:** 2026-11-13

**Authors:** Elena Sophie Hentschke, Alexandros Rahn, Anne Treier, Vivien Dütemeyer, Lena Steinkasserer, Peter Hillemanns, Anna-Lena Biermann, Lena Radomsky, Constantin von Kaisenberg, Lars Brodowski

**Affiliations:** 1 Department of Obstetrics and Gynecology, Hannover Medical School, Hannover, Germany; 2 Department of Pediatric Pulmonology, Allergology and Neonatology, Hannover Medical School, Hannover, Germany

**Keywords:** Preterm prelabor rupture of membranes, Risk Assessment, C-reactive protein, Leukocyte count, Chorioamnionitis, Perinatal outcome, Latency period

## Abstract

**Objective:**

Preterm prelabor rupture of membranes (PPROM) is associated with significant perinatal morbidity and mortality. Clinical management is typically based on inpatient monitoring, although the optimal duration and the potential role of outpatient care remain unclear.

**Methods:**

In this retrospective cohort study, all women with PPROM between 15 + 0 and < 37 + 0 weeks’ gestation managed as inpatients at a tertiary center (07/2019-04/2024) were included. The primary outcome was a composite severe adverse perinatal outcome (SAPO), comprising fetal or neonatal death, neonatal sepsis, intrauterine inflammation or infection, and major obstetric complications. Time-dependent risk was analyzed using latency stratification and landmark analyses. Independent predictors were identified via multivariable logistic regression. Longitudinal clinical and laboratory changes in the five days before delivery were assessed, and predictive performance was evaluated using area under the receiver operating characteristic curve (AUC).

**Results:**

A total of 300 women were included, of whom 46 (15.3%) experienced SAPO. Lower gestational age at PPROM, non-cephalic presentation, and estimated fetal weight below the 10th percentile were independently associated with SAPO, while inflammatory markers at admission showed no statistically significant independent association. The highest adjusted odds of SAPO were observed in the 6–7-day latency group (adjusted OR 6.10, 95% CI 1.45–25.62), although this subgroup comprised only 11 pregnancies. No statistically significant increase was detected in the group with latency exceeding seven days. Longitudinal increases in C-reactive protein (CRP) and leukocyte levels preceded delivery in complicated cases, but predictive performance remained limited (AUC 0.59–0.61).

**Conclusion:**

These findings highlight the importance of gestational age, fetal condition, and dynamic selection during expectant management of PPROM. Prospective studies are needed to evaluate individualized monitoring strategies and the potential role of outpatient management in carefully selected patients.

**Supplementary Information:**

The online version contains supplementary material available at https://doi.org/10.1186/s12884-026-10211-3.

## Introduction

Preterm prelabor rupture of membranes (PPROM) complicates approximately 3% of pregnancies and accounts for a substantial proportion of preterm births, contributing significantly to perinatal morbidity and mortality [1, 2]. The clinical course following PPROM is highly variable and largely determined by the balance between risks associated with prematurity and those related to intrauterine infection and acute obstetric complications [3].

The major risks for the fetus and neonate include intraamniotic infection and inflammation (triple I), placental abruption, umbilical cord complications, and neonatal sepsis, in addition to complications of prematurity [4]. At the same time, prolongation of pregnancy is generally associated with improved neonatal outcomes due to advancing gestational age [5]. However, this benefit must be weighed against the potential for maternal and fetal complications during the latency period.

Current management strategies typically involve expectant inpatient monitoring, including administration of antenatal corticosteroids, antibiotic therapy, and close clinical surveillance [6]. Nevertheless, prolonged hospitalization is associated with psychological stress, increased healthcare costs, and potential nosocomial risks [7, 8]. As a result, outpatient management has been proposed as a potential alternative in selected cases: several studies have attempted to identify criteria for safe outpatient management, suggesting that various clinical factors, including gestational age, fetal presentation, and markers of infection, may be associated with adverse outcomes [9–11]. However, these criteria remain heterogeneous, and there is currently no consensus on how to reliably stratify patients according to risk. Importantly, most existing approaches rely on static parameters assessed at admission and do not account for the dynamic evolution of clinical status during the latency period.

In addition, although close clinical and laboratory monitoring is recommended, the predictive value of commonly used parameters such as leukocyte count, CRP, and maternal temperature remains limited. To date, no reliable and widely accepted method exists to identify patients at low risk who might safely transition to outpatient care after an initial period of stabilization. This underscores the need for improved approaches to risk stratification that consider changes in clinical status over time. Therefore, a better understanding of time-dependent risk patterns and dynamic changes in clinical parameters following PPROM is needed.

The aim of this study was to evaluate time-dependent risk of severe adverse perinatal outcomes (SAPO) following PPROM and to identify clinical predictors, including dynamic changes in routinely collected parameters. We further sought to explore whether longitudinal assessment of clinical markers may improve risk stratification and inform future management strategies. Although outpatient management was not implemented in the present cohort, its potential role in selected low-risk patients requires further evaluation in prospective studies.

## Methods

This retrospective cohort study included all women with preterm prelabor rupture of membranes between 15 + 0 and < 37 + 0 weeks of gestation who were managed at our institution between July 2019 and April 2024. Consecutive cases were identified from a structured clinical database to minimize selection bias. Pregnancies with major fetal malformations and cases with missing information on the primary outcome were excluded. All patients were managed as inpatients according to a standardized institutional protocol. Following PPROM, intravenous aminopenicillin therapy was initiated immediately and continued for at least seven days, with further treatment guided by laboratory findings. Before 34 + 0 weeks of gestation, antenatal corticosteroids were routinely administered as two intramuscular doses of betamethasone (12 mg each) 24 h apart. During corticosteroid administration, intravenous tocolysis with atosiban or a beta-2 sympathomimetic was used. Expectant management was continued in the absence of maternal or fetal indications for delivery. Gestational age at PPROM and at delivery were extracted from the database. Latency was defined as the interval between rupture of membranes and delivery and was analyzed in days. The sample size was determined pragmatically by the number of consecutive pregnancies meeting the eligibility criteria during the predefined retrospective study period. Accordingly, the study was considered exploratory, and the available sample size was used to investigate potential associations between clinical characteristics, latency duration, and adverse perinatal outcomes. Residual amniotic fluid volume and amniotic fluid characteristics, including meconium staining, were not systematically available as standardized variables and were therefore not included in the statistical analyses.

The primary outcome was a composite severe adverse perinatal outcome (SAPO), defined as the occurrence of at least one of the following complications: fetal or early neonatal death, neonatal sepsis, suspected intra-amniotic infection or inflammation, antepartum hemorrhage, placental abruption, umbilical cord prolapse, or pathological fetal Doppler findings requiring delivery. Fetal or neonatal death was defined as intrauterine fetal death after PPROM or neonatal death within the first seven days of life. Neonatal sepsis included both early-onset and late-onset sepsis diagnosed during neonatal hospitalization. Diagnosis was based on clinical findings, with or without microbiological confirmation by positive neonatal blood cultures. Both culture-proven and clinically diagnosed cases were included. Suspected triple I was defined according to the German AWMF guideline (015–025, version 5.0, September 2022) as maternal fever of unknown origin accompanied by at least one of the following: fetal tachycardia exceeding 160 beats/min for more than 10 min, maternal leukocytosis exceeding 15,000/µL in the absence of corticosteroid administration, or purulent cervical discharge. Histopathological placental examination or biochemical analysis of amniotic fluid was not required for diagnosis. Pathological fetal Doppler findings were defined as gestational age-dependent abnormalities of the umbilical artery, middle cerebral artery, ductus venosus, or their combined assessment that constituted an indication for delivery according to the applicable German AWMF guideline (015/080) [12].

For time-dependent analyses, latency was defined as the interval between PPROM and delivery. Neonatal outcomes diagnosed after birth, including neonatal sepsis and early neonatal death, were assigned to the day of delivery rather than the actual postnatal day of diagnosis. Accordingly, the time-dependent analyses reflect the distribution of pregnancies associated with adverse outcomes according to latency duration, rather than the exact chronological occurrence of all individual complications.

Secondary outcomes included respiratory morbidity, low arterial umbilical cord pH (< 7.1), low Apgar score at 5 min (< 7), duration of neonatal hospitalization, and latency duration. Respiratory morbidity was defined as the occurrence of at least one of the following: oxygen supplementation, resuscitation at birth, mask ventilation, endotracheal intubation, surfactant administration, need for continuous positive airway pressure (CPAP), high-flow nasal cannula (HFNC), or bronchopulmonary dysplasia.

Clinical variables assessed at admission included maternal age, pre-pregnancy body mass index, parity, gestational age at PPROM, cervical length measured by transvaginal ultrasound, fetal presentation, estimated fetal weight percentile, inflammatory markers including leukocyte count and CRP, and vaginal microbiological findings. These variables were grouped into maternal, fetal, and laboratory parameters for analysis. Cervical length was analyzed using predefined cut-offs of < 25 mm and < 15 mm. Estimated fetal weight below the 10th percentile was defined as an ultrasound-estimated fetal weight below the gestational age-specific 10th percentile at the initial assessment following PPROM. This variable was used as a sonographic measure of fetal size and was not considered equivalent to a confirmed diagnosis of fetal growth restriction. Non-cephalic presentation included breech and transverse lie.

Distributional assumptions were assessed using the Shapiro-Wilk test. Continuous variables are presented as median and interquartile range, and categorical variables as counts and percentages. Group comparisons between women with and without severe adverse perinatal complications were performed using Student’s t test or the Mann-Whitney U test for continuous variables and the χ² test or Fisher’s exact test for categorical variables, as appropriate.

For time-dependent analyses, latency was categorized into four groups (0–2, 3–5, 6–7, and > 7 days) to reflect clinically relevant intervals following PPROM. Associations between latency groups and SAPO were assessed using logistic regression models adjusted for gestational age at PPROM. Landmark analyses were performed at days 3, 5, and 7 after PPROM, including only women who remained pregnant and had not experienced the primary outcome before the respective time point; this approach was used to partially account for time-dependent bias and differences in follow-up duration, with day 1 at admission used as the reference. To identify independent predictors of SAPO, multivariable logistic regression was performed including gestational age at PPROM, cervical length (< 25 mm and < 15 mm), fetal presentation, estimated fetal weight below the 10th percentile, positive vaginal swab, leukocyte count > 15.000/µl, and CRP > 5 mg/L at admission. Missing data were not imputed. Multivariable logistic regression was performed using complete cases with available data for all included covariates. The multivariable model included 63 pregnancies, of which 16 experienced SAPO. Given the limited number of outcome events, the number of variables included in the multivariable model was restricted to mitigate the risk of overfitting and were considered exploratory. Results are reported as adjusted odds ratios with 95% confidence intervals.

Time-to-event analyses were performed using Kaplan-Meier methods to assess the cumulative incidence of SAPO and their individual components after PPROM. Latency in days after PPROM was used as the time scale, and cumulative event probability was plotted for the composite outcome as well as for fetal/neonatal death, neonatal sepsis, triple I, antepartum hemorrhage, placental abruption, umbilical cord prolapse, and pathological Doppler findings.

For longitudinal analyses, day-to-day changes (Δ) were calculated for each patient as the difference between consecutive daily measurements during the five days preceding delivery. Median Δ-values were determined for each time point and compared between uncomplicated and complicated cases using the Mann-Whitney U test. Predictive performance of these Δ-values was assessed by receiver operating characteristic analysis, and the area under the curve was calculated. All longitudinal analyses were considered exploratory. Additional subgroup analyses were performed for triple I. Statistical analyses were performed using GraphPad Prism 9 software (GraphPad Software, Boston, Massachusetts, USA) and IBM SPSS Statistics 28 (IBM SPSS Software, Armonk, NY, USA). A two-sided p-value < 0.05 was considered statistically significant. Ethical approval was obtained from the local ethics committee (12377-BO-K-2026).

## Results

A total of 300 women with PPROM were included in the final analysis. Cases with severe fetal malformations (*n* = 19) and pregnancies complicated by preeclampsia (*n* = 3) were excluded prior to analysis. Overall, 46 women (15.3%) experienced a SAPO. The individual components of the composite outcome included neonatal sepsis in 12 cases (4.0%), fetal or neonatal death in 5 cases (1.7%), and acute obstetric complications such as triple I in 21 cases (7.0%), antepartum hemorrhage in 2 cases (0.7%), placental abruption in 3 cases (1.0%), umbilical cord prolapse in 3 cases (1.0%), and pathological Doppler findings in 3 cases (1.0%).

### Baseline characteristics

Baseline maternal characteristics were comparable between women with and without SAPO (Table 1). In contrast, gestational age at PPROM was lower in women with SAPO (*p* < 0.001) and administration of antenatal corticosteroids was more frequent in this group (69.6% vs. 26.8%, *p* < 0.001).

**Table 1:** Characteristics at admission, birth and postpartal. Data are presented as median (interquartile range), or n (%), as appropriate. Percentages were calculated using the number of available observations for each variable. Latency was defined as the time interval between PPROM and delivery. Non-cephalic presentation includes breech and transverse lie. Respiratory morbidity is defined as the occurrence of at least one of the following: requirement for oxygen supplementation, resuscitation at birth, mask ventilation, endotracheal intubation, administration of surfactant, need for continuous positive airway pressure or high-flow nasal cannula, or the presence of bronchopulmonary dysplasia. CRP = C-reactive protein; PPROM = preterm prelabor rupture of membranes; SAPO = severe adverse perinatal outcome

	PPROM without SAPO (*n* = 254)	PPROM with SAPO (*n* = 46)	*p*-value
Maternal age (years)	33.0 [29.0–36.0]	33.5 [29.0–36.0]	0.689
Pre-pregnancy Body mass index (BMI)	24.9 [22.0-29.3]	22.7 [20.6–26.9]	0.069
Parity	0.0 [0.0–1.0]	0.5 [0.0–1.0]	0.614
Administration of antenatal steroids	68/254 (26.8%)	32/46 (69.6%)	**< 0.001**
Gestational age at PPROM (weeks) < 24 24–26 26–28 28–32 32–34 > 34	5 (35.7%) 4 (57.1%) 5 (62.5%) 25 (67.6%) 30 (83.3%) 185 (93.4%)	9 (64.3%) 3 (42.9%) 3 (37.5%) 12 (32.4%) 6 (16.7%) 13 (6.6%)	**< 0.001**
Cervical length < 25 mm (ultrasound)	43/74 (58.1%)	12/28 (42.9%)	0.19
Cervical length < 15 mm (ultrasound)	16/74 (21.6%)	7/28 (25.0%)	0.79
**Infection-related parameters at admission**			
Leukocytes > 15.000/µl	23/200 (11.5%)	5/34 (14.7%)	0.57
CRP > 5 mg/l	91/199 (45.7%)	19/34 (55.9%)	0.35
Maternal fever (> 39.0° or 2x > 38.0° in 30 min)	0/254 (0.0%)	0/46 (0.0%)	N/A
Positive vaginal sample	44/88 (50.0%)	18/32 (56.3%)	0.68
**Initial ultrasound**			
Cephalic presentation	224/254 (88.2%)	26/46 (56.5%)	**< 0.001**
Estimated fetal weight under 10th percentile	9/178 (5.1%)	8/33 (24.2%)	**< 0.001**
**Birth**			
Gestational age at birth (weeks)	35.9 [34.6–36.7]	31.5 [27.8–35.1]	**< 0.001**
Latency period (days)	1.0 [0.0–3.0]	4.0 [1.0-8.8]	**0.004**
Mode of delivery: Cesarean section	48/254 (18.9%)	31/46 (67.4%)	**< 0.001**
**Neonatal outcome**			
Neonatal birth weight (grams)	2543 [2100–3000]	1659 [900–2400]	**< 0.001**
APGAR < 7 at 5 min	7/254 (2.8%)	12/46 (26.1%)	**< 0.001**
Umbilical arterial pH < 7.1	8/254 (3.1%)	0 (0.0%)	0.470
**Complications**			
Respiratory morbidity	42/254 (16.5%)	20/46 (43.5%)	**< 0.001**
Neonatal sepsis	0 (0.0%)	12 (26.1%)	**< 0.001**
Duration of hospitalization (days)	7.0 [4.0–13.0]	31.5 [8.5–54.0]	**< 0.001**

At admission, neither cervical length < 25 mm (42.9% vs. 58.1%, *p* = 0.19) nor < 15 mm (25.0% vs. 21.6%, *p* = 0.79) differed significantly between women with and without SAPO. Similarly, no significant differences were observed for leukocyte count > 15,000/µL (14.7% vs. 11.5%, *p* = 0.57), CRP > 5 mg/L (55.9% vs. 45.7%, *p* = 0.35), or positive vaginal microbiological samples (56.3% vs. 50.0%, *p* = 0.68). No cases of maternal fever were observed.

Fetal characteristics differed between groups. Non-cephalic presentation was more frequent in the SAPO group (43.5% vs. 11.8%, *p* < 0.001), and estimated fetal weight below the 10th percentile was significantly more common (24.2% vs. 5.1%, *p* < 0.001).

### Perinatal outcomes

Women with SAPO delivered at earlier gestational age (31.5 vs. 35.9 weeks, *p* < 0.001) and had longer latency periods (4.0 vs. 1.0 days, *p* = 0.004). Cesarean delivery was more frequent in the SAPO group (67.4% vs. 18.9%, *p* < 0.001). Neonatal outcomes were less favorable in the SAPO group, including lower birth weight (1659 g vs. 2543 g, *p* < 0.001), increased rates of low Apgar score at 5 min (26.1% vs. 2.8%, *p* < 0.001), higher rates of respiratory morbidity (43.5% vs. 16.5%, *p* < 0.001), and neonatal sepsis (26.1% vs. 0%, *p* < 0.001). Duration of neonatal hospitalization was longer in this group (31.5 vs. 7.0 days, *p* < 0.001).

### Time-dependent risk of adverse outcome and multivariable analysis

**Fig. 1:**
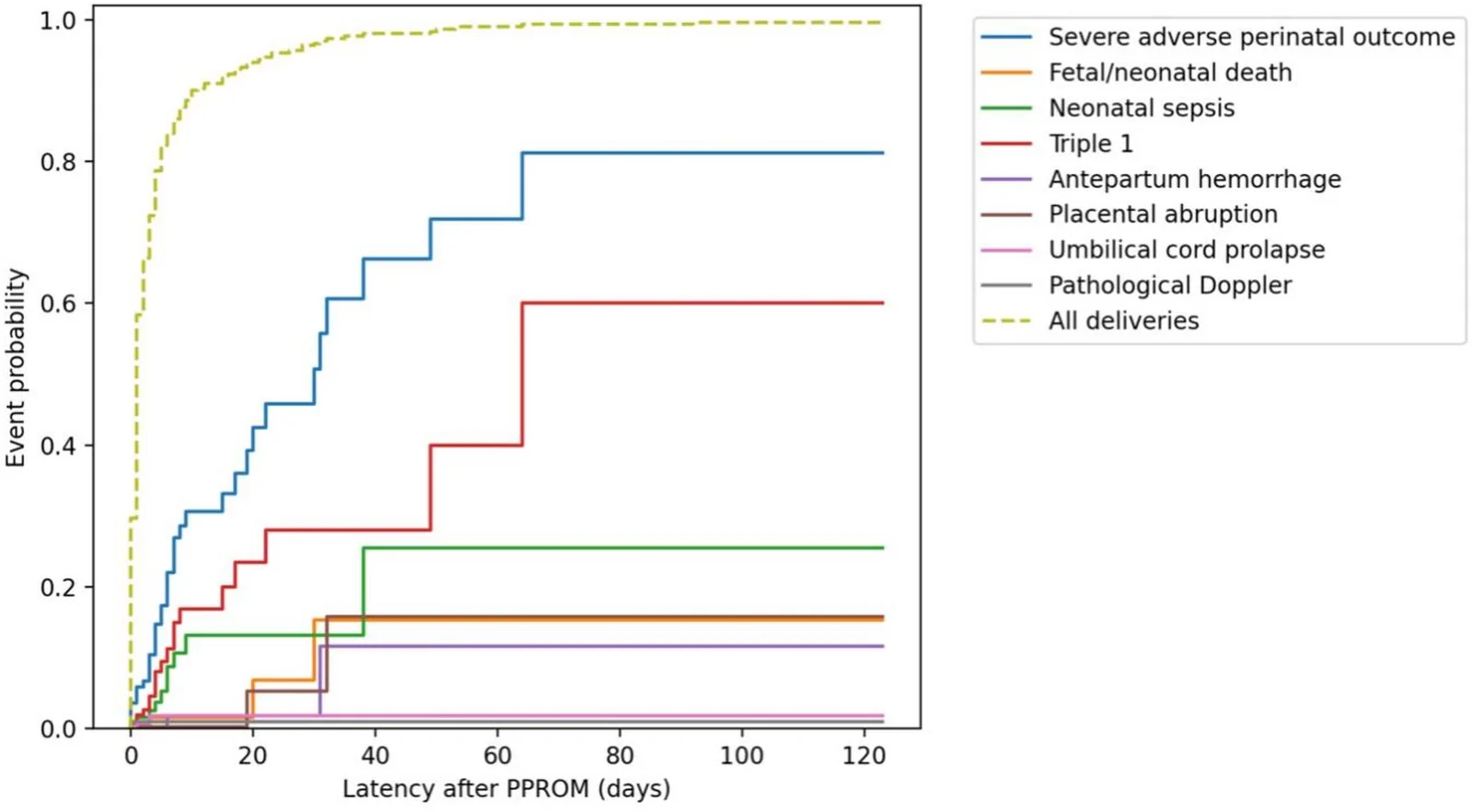
Cumulative incidence of SAPO and individual components following PPROM. The figure shows the cumulative incidence of severe adverse perinatal outcome and its individual components over time following PPROM. The x-axis represents latency in days after PPROM, and the y-axis represents cumulative event probability. Time was measured from PPROM to the respective event. Pregnancies delivered without the event of interest were censored at delivery. PPROM = preterm prelabor rupture of membranes, SAPO = severe adverse perinatal outcome

The cumulative incidence of SAPO over time following PPROM is shown in Fig. 1. Adverse events occurred predominantly within the early period after membrane rupture.

In latency-stratified analyses (Table 2), the proportion of SAPO varied across latency categories, with the highest proportion observed in the 6-7-day interval (54.5%; adjusted OR 6.10 [1.45–25.62], *p* = 0.013). This estimate was based on a small number of cases in this interval (*n* = 11). No significant association was observed beyond 7 days (> 7 days: OR 0.97 [0.32–2.99], *p* = 0.964).

**Table 2:** Time-dependent risk and predictors of SAPO following PPROM. Latency was defined as the interval between PPROM and delivery. Latency groups were analyzed using logistic regression models adjusted for gestational age at PPROM. Landmark analyses were performed at days 3, 5, and 7 after PPROM, including only women who remained pregnant and had not experienced the primary outcome prior to the respective time point; these analyses were adjusted for gestational age at PPROM, with day 1 (at admission) as the reference. Multivariable analysis was performed using logistic regression including gestational age at PPROM (continuous), cervical length (< 25 mm and < 15 mm), fetal presentation (cephalic vs. non-cephalic), estimated fetal weight below the 10th percentile, positive vaginal swab, leukocyte count > 15.000/µl, and C-reactive protein > 5 mg/L at admission. Results are presented as adjusted odds ratios (OR) with 95% confidence intervals (CI). CRP = C-reactive protein; PPROM = preterm prelabor rupture of membranes; SAPO = severe adverse perinatal outcome

Analysis	*N*	SAPO, *n* (%)	Adjusted OR (95% CI)	*p*-value
**Latency groups**				
0–2 days	199	17 (8.5%)	Reference	-
3–5 days	48	10 (20.8%)	1.70 [0.68–4.28]	0.260
6–7 days	11	6 (54.5%)	6.10 [1.45–25.62]	**0.013**
> 7 days	42	13 (31.0%)	0.97 [0.32–2.99]	0.964
**Landmark analysis**				
Day 1 (at admission)	300	-	Reference	-
Day 3 (still pregnant)	83	25 (30.1%)	1.40 [0.75–2.63]	0.296
Day 5 (still pregnant)	53	19 (35.8%)	1.38 [0.66–2.88]	0.395
Day 7 (still pregnant)	42	13 (31.0%)	0.94 [0.40–2.19]	0.888
**Multivariable predictors of SAPOs**				
Gestational age at PPROM (per week)			**0.81 [0.74–0.89]**	**< 0.001**
Cervical length < 25 mm ad admission			0.96 [0.29–3.17]	0.945
Cervical length < 15 mm ad admission			1.92 [0.43–8.54]	0.392
Cephalic presentation			**0.21 [0.09–0.50]**	**< 0.001**
Estimated fetal weight under 10th percentile			**7.54 [2.19–25.94]**	**0.001**
Positive vaginal sample ad admission			0.96 [0.38–2.42]	0.934
Leukocytes > 15.000/µl ad admission			0.81 [0.22–3.07]	0.761
CRP > 5 mg/l ad admission			1.65 [0.72–3.75]	0.236

In landmark analyses including only women who remained pregnant at predefined time points, no significant differences in risk were observed at days 3, 5, or 7 compared to baseline (all *p* > 0.29).

Importantly, latency-stratified analyses and landmark analyses address different aspects of the association between latency duration and adverse outcome. Whereas latency categories were defined according to the total interval between PPROM and delivery, landmark analyses were restricted to pregnancies remaining ongoing and event-free at predefined time points. Consequently, the populations contributing to later time points were increasingly selected. The elevated odds observed in the 6–7-day latency group should be interpreted cautiously because of the small sample size (*n* = 11) and the resulting wide confidence interval.

In multivariable analysis, lower gestational age at PPROM was independently associated with SAPO (adjusted OR 0.81 per week [0.74–0.89], *p* < 0.001). Cephalic presentation was associated with lower risk (OR 0.21 [0.09–0.50], *p* < 0.001), whereas estimated fetal weight below the 10th percentile was associated with increased risk (OR 7.54 [2.19–25.94], *p* = 0.001). Cervical length, vaginal microbiological findings, and inflammatory markers at admission (leukocytes and CRP) were not independently associated with SAPO.

### Longitudinal changes in clinical parameters

**Fig. 2:**
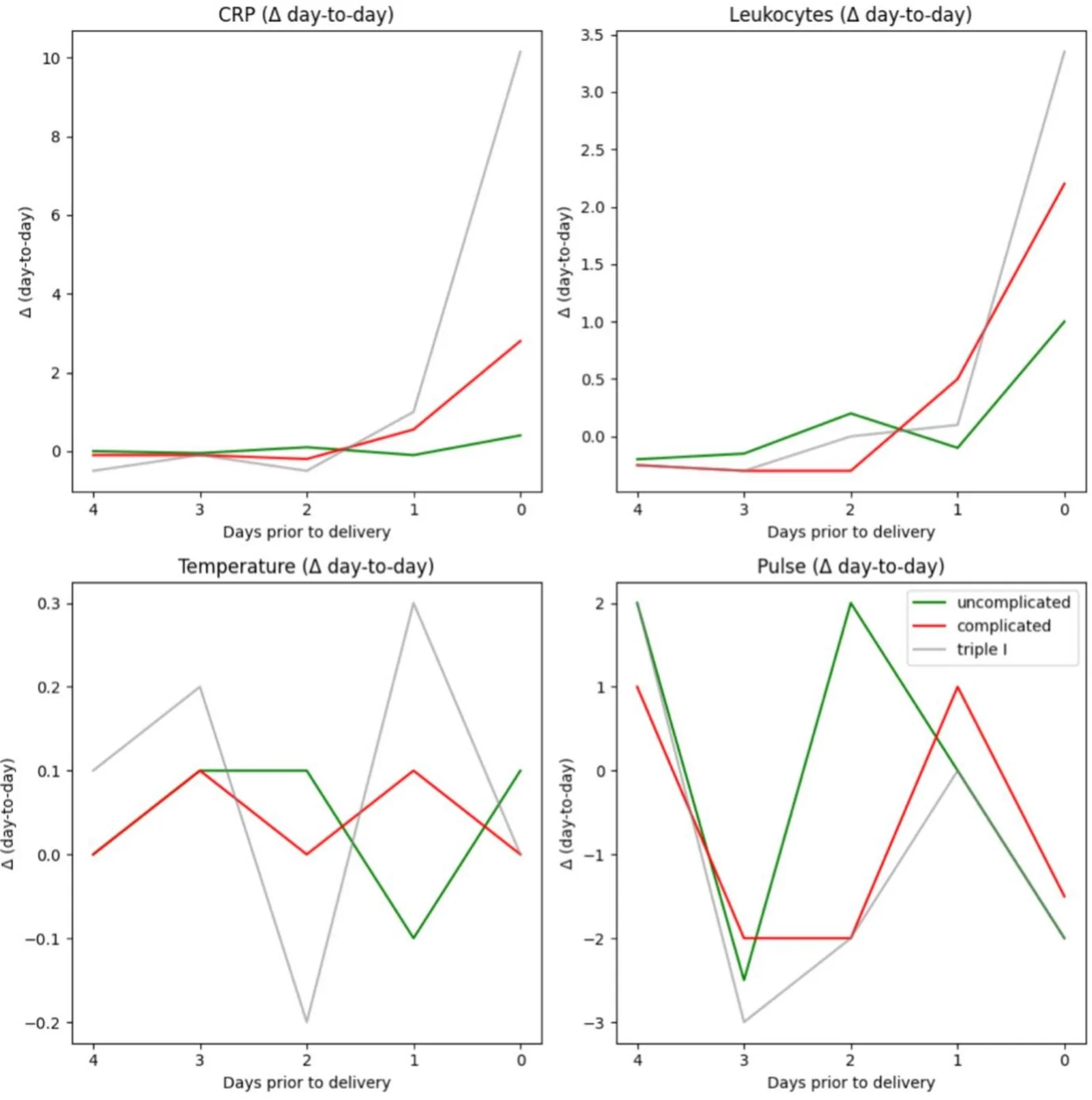
Day-to-day changes in clinical parameters prior to delivery in PPROM. Median daily changes (Δ) in CRP, leukocyte count, temperature, and pulse during the five days preceding delivery for uncomplicated and complicated (SAPO) cases, as well as for cases of triple I. The x-axis represents days prior to delivery (day 0 = delivery). CRP = C-reactive protein; PPROM = preterm prelabor rupture of membranes; SAPO = severe adverse perinatal outcome; triple I = intrauterine inflammation or infection

Longitudinal analyses of clinical and laboratory parameters in the five days preceding delivery are shown in Fig. 2 (day-to-day changes) and Supplemental Fig. 1 (absolute values). In cases with SAPO, CRP levels increased prior to delivery, particularly in cases of triple I. Leukocyte counts showed less pronounced changes, while temperature and pulse showed minimal variation.

In quantitative analyses (Table 3), daily increases in CRP were higher in complicated compared to uncomplicated cases (+ 2.8 vs. +0.4), although this difference did not reach statistical significance (*p* = 0.08). Leukocyte changes showed a similar pattern (+ 2.2 vs. +1.0, *p* = 0.135). Temperature and pulse showed no relevant differences. The discriminative ability of day-to-day changes was limited, with AUC values of 0.59 for CRP and 0.61 for leukocytes.

**Table 3:** Predictive value of day-to-day changes in clinical parameters for SAPO. Day-to-day changes (Δ) represent the difference between consecutive measurements. Median Δ-values and their association with adverse outcomes are shown. Predictive performance was evaluated using AUC (area under the curve). CRP = C-reactive protein; PPROM = preterm prelabor rupture of membranes; SAPO = severe adverse perinatal outcome

Parameter	Δ (without SAPO)	Δ (with SAPO)	*p*-value	AUC
CRP	+ 0.4	+ 2.8	0.080	0.59
Leukocytes	+ 1.0	+ 2.2	0.135	0.61
Temperature	+ 0.1	0.0	0.408	0.46
Pulse	-2.0	-1.5	0.828	0.47

## Discussion

In this retrospective cohort study of women with PPROM, we observed that SAPO occurred in approximately 15% of cases and were strongly associated with gestational age at PPROM, non-cephalic presentation, and estimated fetal weight below the 10th percentile. These findings are consistent with previous studies demonstrating that gestational age is the most important determinant of neonatal outcome following PPROM [13, 14].

Management of PPROM is fundamentally guided by the assumption that prolongation of pregnancy improves neonatal outcome but may increase infectious or obstetric risk. Current guidelines emphasize gestational age as the dominant determinant of management, while recommending continuous inpatient monitoring because of the perceived unpredictability of clinical deterioration [5, 15]. However, the temporal structure of risk during the latency period has rarely been analyzed beyond descriptive latency distributions.

Our findings suggest that adverse perinatal outcomes following PPROM are concentrated during the early clinical course rather than increasing consistently with prolonged latency, in line with reports that more than half of women deliver within seven days after PPROM [16]. However, time-dependent associations must be interpreted in light of dynamic selection. Latency-stratified analyses compare pregnancies according to their eventual latency duration, whereas landmark analyses include only pregnancies that remain ongoing and free of previously observed adverse events at each time point. Consequently, later groups represent selected pregnancies with a more favorable preceding clinical course [16]. Although the highest adjusted odds of SAPO were observed at 6–7 days, this subgroup included only 11 pregnancies. Neither this uncertain estimate nor the absence of a significant association beyond seven days establishes a distinct risk peak, declining risk, or the safety of prolonged pregnancy.

Early complications may partly reflect inflammatory processes already present at membrane rupture, consistent with proposed roles of intra-amniotic inflammation and oxidative stress in PPROM [17, 18]. However, these mechanisms were not directly investigated and remain speculative. Overall, our results highlight the importance of accounting for survivor selection when interpreting latency-dependent outcomes. They do not establish a safe latency threshold or support outpatient management. Prospective studies are needed to evaluate individualized monitoring and management strategies. Fetal condition emerged as an additional determinant of risk. In our cohort, estimated fetal weight below the 10t^h^ percentile and non-cephalic presentation were associated with adverse outcome. Estimated fetal weight below the 10th percentile is well established to be associated with adverse perinatal outcome in preterm infants [17, 18], suggesting that reduced fetal reserve may contribute to increased vulnerability also in PPROM. It remains unclear whether this reflects a PPROM-specific effect or the underlying risk associated with fetal growth restriction itself. For non-cephalic presentation, previous data suggest an association with increased neonatal mortality in PPROM, although the underlying mechanisms remain unclear [19]. Together, these findings support the concept that fetal vulnerability may contribute to risk beyond inflammatory processes and latency duration.

The limited prognostic value of leukocyte count and CRP at admission (and the modest discriminative ability of their day-to-day changes) align with prior literature demonstrating limited diagnostic accuracy of maternal CRP for intraamniotic inflammation in PPROM [20]. In our cohort, inflammatory markers at admission were not associated with adverse outcome, and short-term changes showed only modest performance (AUC ~ 0.6). Although leukocyte counts and CRP tended to increase prior to delivery in pregnancies with adverse outcomes, these changes did not reach statistical significance. Given the limited number of SAPO events and the exploratory nature of our analyses, the absence of statistically significant associations should not be interpreted as evidence that these markers lack clinical or prognostic value. Rather, our findings suggest that conventional inflammatory markers are insufficient as standalone tools for predicting the heterogeneous composite SAPO endpoint. Importantly, their limited performance in predicting this composite outcome does not exclude their potential usefulness in detecting evolving infection or supporting clinical decisions when interpreted alongside maternal symptoms, temperature, fetal monitoring, and other relevant clinical findings. This distinction is particularly important because the SAPO endpoint encompasses both infectious and non-infectious complications, which may not be equally reflected by systemic inflammatory markers. Accordingly, our findings underscore the limitations of relying on isolated laboratory measurements for risk assessment [20] and highlight the need for more comprehensive approaches that integrate clinical information with longitudinal changes over time.

Current management strategies rely on expectant inpatient monitoring due to concerns regarding infection, fetal compromise, and unpredictable deterioration [21, 22]. However, these approaches are largely based on clinical observation and laboratory parameters, whose predictive value appears limited, as also reflected in our findings. This may partly explain why optimal monitoring strategies remain difficult to define. Previous studies have suggested that outpatient management may be feasible in carefully selected patients without increasing adverse outcomes and may even be associated with prolonged latency and improved neonatal outcomes [9–11]. However, these approaches typically rely on static selection criteria assessed at admission or after a short stabilization period, such as gestational age, cervical length, fetal presentation, and absence of infection. In this context, our findings suggest that early clinical course may provide more informative signals for risk stratification than single baseline measurements obtained at admission and may help to identify patients who remain stable after an initial phase.

Taken together, our findings suggest that latency duration alone may not adequately reflect risk after PPROM. Instead, risk appears to be influenced by gestational age at rupture, fetal condition, and early clinical course.

Although our findings may have implications for the development of individualized monitoring strategies following PPROM, their relevance to step-down or outpatient management remains strictly hypothesis-generating. All pregnancies in the present cohort were managed in an inpatient setting, and neither the feasibility nor the safety of alternative care settings was evaluated. Furthermore, pregnancies remaining clinically stable over time represent a dynamically selected subgroup, and the absence of a statistically significant association with adverse outcomes at later time points cannot be interpreted as evidence that reduced monitoring intensity or outpatient care would be safe. Accordingly, our results should not be used to support changes in the setting or intensity of clinical surveillance. Prospective studies incorporating predefined selection criteria, standardized monitoring protocols, and appropriate maternal and neonatal safety endpoints are required to determine whether carefully selected women with PPROM could safely receive step-down or outpatient care.

From a clinical perspective, our findings do not establish a seven-day threshold for safe prolongation of pregnancy after PPROM. Continued expectant management should remain individualized, based on gestational age, maternal and fetal condition, and the absence of indications for delivery, particularly suspected intra-amniotic infection or fetal compromise. Close maternal and fetal surveillance remains essential, including clinical assessment, fetal monitoring, and relevant laboratory parameters. As these management strategies were not directly evaluated in our inpatient cohort, their implications for prolonged expectant or outpatient care remain hypothesis-generating and require prospective validation. This study has several strengths. It includes a well-characterized cohort of women with PPROM across a wide gestational age range, managed within a standardized clinical setting. The use of time-dependent analyses, including latency stratification and landmark approaches, allowed for a more nuanced assessment of risk over time and helped to account for differences in follow-up duration. In addition, the integration of longitudinal clinical data provides insight into dynamic changes preceding delivery.

Nevertheless, several limitations should be acknowledged. The retrospective design limits causal inference and may be subject to residual confounding. The number of outcome events was relatively limited, restricting the number of variables included in multivariable analyses and potentially affecting model stability. Some latency subgroups, particularly the 6–7-day interval, included small numbers of patients, limiting precision of estimates. Furthermore, components of the composite outcome may be influenced by clinical decision-making, introducing potential management-related bias. An important limitation is the heterogeneous SAPO composite, which combines fetal and neonatal outcomes with obstetric complications and management-dependent findings. Pathological fetal Doppler findings may reflect indications for delivery rather than adverse neonatal outcomes. However, an exploratory sensitivity analysis excluding this component reduced the SAPO rate only slightly (15.3% to 14.3%), while the main latency-related associations remained qualitatively consistent, including increased odds at 6–7 days and no statistically significant association beyond seven days. These estimates remain imprecise because of the small number of events.

Furthermore, neonatal sepsis and early neonatal death were assigned to the day of delivery, meaning that our analyses describe associations with latency rather than the exact timing of all SAPO components. The limited number of complete cases and SAPO events in the multivariable analysis may have affected the precision and stability of the adjusted estimates. Incomplete clinical and laboratory data represent an additional limitation of the retrospective design. Future prospective studies should distinguish antepartum events, delivery indications, and postnatal outcomes. Lastly, residual amniotic fluid volume and abnormal amniotic fluid characteristics were not systematically available, potentially limiting the assessment of additional indicators of fetal compromise or intra-amniotic infection. As this was a single-center study conducted at a tertiary care institution, the generalizability of our findings to other clinical settings may be limited. Finally, outpatient management was not implemented in this cohort, and conclusions regarding its safety remain hypothetical.

## Conclusion

In this retrospective cohort of women with PPROM, adverse perinatal outcomes were associated with gestational age at membrane rupture, fetal condition, and latency duration. The interpretation of time-dependent associations requires consideration of the dynamic selection of pregnancies remaining ongoing and clinically stable during expectant management. In particular, the absence of a statistically significant association with prolonged latency should not be interpreted as evidence of low absolute risk or as support for outpatient management without further validation. Commonly used inflammatory markers showed limited predictive performance. Future prospective studies are needed to develop and validate individualized, time-dependent risk stratification strategies and to evaluate the safety of alternative monitoring approaches in carefully selected patients.

## Supplementary Material

Below is the link to the electronic supplementary material.

Supplementary Material 1

## Data Availability

The datasets generated and analyzed during this study are not publicly available due to patient confidentiality regulations but are available from the corresponding author upon reasonable request.
