# Supplementary material for: Not all days are equal after preterm prelabor rupture of membranes: a time-dependent analysis of adverse perinatal outcomes
Source: BMC Pregnancy Childbirth. 2026 Nov 13;26:1322. doi: 10.1186/s12884-026-10211-3 (PMC13760755; doi:10.1186/s12884-026-10211-3)
Supplement: Supplementary file 1 [file 12884_2026_10211_MOESM1_ESM.docx]

Supplemental Figure 1. Longitudinal changes in clinical parameters prior to delivery in PPROM.

Median values of CRP, leukocyte count, temperature, and pulse are shown for uncomplicated and complicated (SAPO) cases, as well as for cases of triple I. The x-axis represents days prior to delivery (day 0 = delivery). CRP = C-reactive protein; PPROM = preterm prelabor rupture of membranes; SAPO = severe adverse perinatal outcome; triple I = intrauterine inflammation or infection.
